# Coexisting *BAG3* Variant and the Anomalous Origin of the Right Coronary Artery Presenting with Recurrent Ventricular Tachycardia

**DOI:** 10.19102/icrm.2023.14051

**Published:** 2023-05-15

**Authors:** Ilsen E. Hernandez, Atul Prakash

**Affiliations:** ^1^Medicine/Cardiology Department, St. Mary’s General Hospital, Passaic, NJ, USA

**Keywords:** Cardiomyopathy, coronary vessel anomaly, genetic disorders, palpitations, ventricular tachycardia

## Abstract

A 49-year-old woman presented with recurrent palpitations and presyncope. Monitoring revealed recurrent non-sustained ventricular tachycardia (VT) episodes. Cardiac catheterization showed the right coronary artery originating from the left coronary cusp. Cardiac computerized tomography revealed the course between the aorta and the pulmonary artery. Despite surgical correction, VT persisted. Genetic testing revealed a rare BCL2-associated athanogene 3 (*BAG3*) variant associated with dilated cardiomyopathy.

## Case presentation

A 49-year-old woman presented with palpitations and light-headedness for 2–3 weeks.

There was no family history of sudden death or premature cardiac disease. The patient’s mother has a history of a neurological disorder with involuntary movements. There was no history of drug or alcohol abuse and no excessive caffeine intake. Her physical examination was unremarkable.

### Investigation

**Figure 1:**
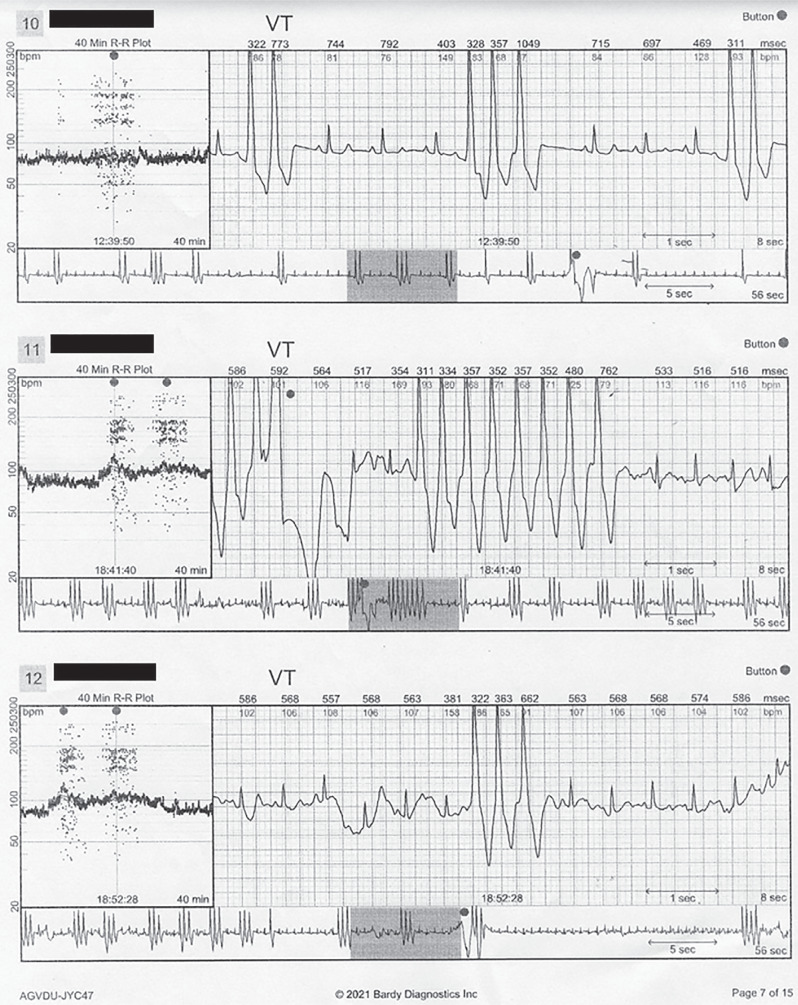
Recurrent unsustained ventricular tachycardia. This event monitor recording demonstrates recurrent unsustained VT. The VT is monomorphic with similar cycle length. *Abbreviation:* VT, ventricular tachycardia.

**Figure 2:**
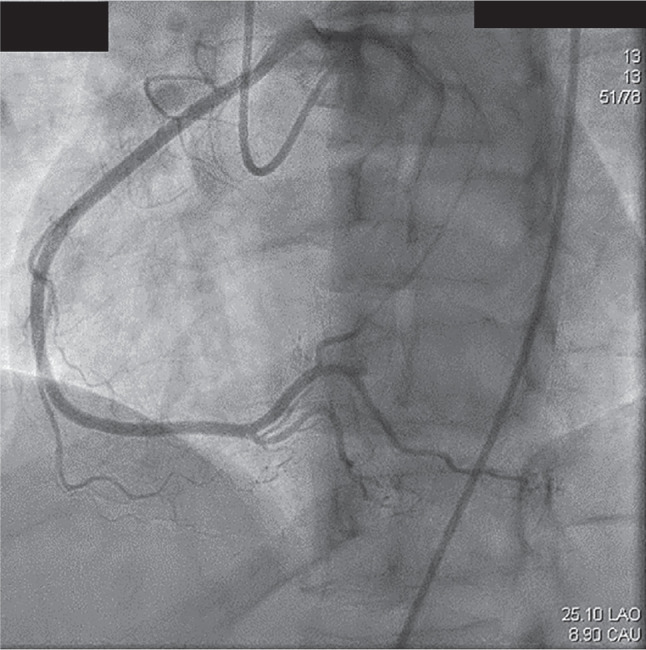
Right coronary artery (RCA) anomaly. Coronary artery angiogram demonstrating an anomalous RCA originating from the left coronary cusp. Contrast is seen in the left coronary system as well. This is a dominant RCA without any significant stenosis.

An electrocardiogram showed normal sinus rhythm with a normal QT interval, no overt pre-excitation, and no evidence of Brugada syndrome. Echocardiography showed preserved left ventricular systolic function and normal chamber dimensions with no valvular abnormalities. Event monitoring for 1 week did not reveal any tachyarrhythmias. A second event monitor was repeated because of the persistence of her symptoms, which showed multiple episodes of non-sustained ventricular tachycardia (VT) **(Figure 1)**. Cardiac catheterization demonstrated an anomalous right coronary artery (RCA) originating from the left coronary cusp **(Figure 2)**. Computerized tomography imaging was performed, which confirmed and delineated the course of the RCA between the aorta and the pulmonary artery.

### Management

The patient underwent surgical correction with reimplantation of the RCA to the right coronary cusp. Despite surgical correction, she continued to have symptomatic non-sustained VT. Monitoring showed daily episodes of VT, 6–24 beats in duration. We administered β-blockers, which had only partial efficacy. Cardiac magnetic resonance imaging was performed to assess for myocardial scarring or infiltrative diseases capable of inducing arrhythmia. There were no discrete scars and no evidence of amyloidosis or sarcoidosis. An electrophysiology study was performed for risk stratification and to consider an ablation strategy if monomorphic sustained VT was induced. Sustained VT was not induced. The patient was initiated on 40 mg of verapamil 3 times daily.

### Follow-up

The patient has been followed up with regularly for >12 months and has remained on verapamil with some improvement. She continues to have symptoms, though they have improved, with shorter and less frequent episodes of VT happing anywhere from daily to 2 times/week. An increase in the dose of verapamil is being considered. During follow-up, the patient reported symptoms of numbness in both of her lower extremities. A diagnosis of peripheral neuropathy is being considered. Genetic testing was performed for a possibility of a primary electrical disorder, and results were negative for catecholaminergic polymorphic VT, Brugada, and long or short QT syndrome. However, a BCL2-associated athanogene 3 (*BAG3*) exon 3 variant was detected. The ventricular arrhythmias have continued despite surgical correction, suggesting a primary electrical abnormality unrelated to the anomalous coronary artery origin. The patient is being closely followed for any increase in symptoms, serial monitoring, and echoes for possible development of a cardiomyopathy and worsening of the VT.

## Discussion

The anomalous origin of the RCA from the left coronary cusp has shown that there are 3 types of anomalous RCA, as follows: (1) a high inter-arterial course between the pulmonary artery and the aorta, (2) a low inter-arterial course between the right ventricular outflow tract and the aorta, and (3) a hypoplastic anomalous RCA orifice.^1^ The incidence of the anomalous origin of the RCA arising from the left coronary cusp that courses between the great vessels varies between 0.026%–0.25%.^2,3^ When this type of anomaly occurs, the RCA is at risk of being compressed during exercise or routine activities, causing angina, malignant arrhythmias, and sudden cardiac death. It has been proposed that, as the pulmonary artery and/or the aorta increases in size, it mechanically compresses the RCA and induces anginal and ischemic symptoms.^1^ Other ischemia-inducing mechanisms have also been observed, including acute angulation at the ostium of the RCA and kinking, an abnormal slit-like opening, and vasospasm of the RCA.^4^ However, no mechanism has been proposed yet to explain arrhythmogenic changes in adult patients with an anomalous RCA originating from the left coronary cusp, and there haven’t been any reported observed cases.

Arrhythmias may eventually occur if an anomalous RCA goes undetected for a long time and presents with arrhythmias in adulthood. This is likely due to repetitive undetected ischemic myocardial damage over the patient’s lifetime.

Furthermore, the detection of the *BAG3* mutation, which is associated with familial cardiomyopathy, has been well studied and established.^5–8^ The majority of *BAG3* mutations identified so far—namely, Pro209Leu (causing myofibrillar myopathy), Arg218Trp (causing dilated cardiomyopathy [DCM]), and 1 of 4 novel mutations associated with fulminant DCM (a large deletion of 17,990 bp removing *BAG3* exons 3–4)—are located within exon 3, indicating the importance of this region for protein activity.^9–11^ Specifically, the mutation causes moderate myocyte hypertrophy and patchy mild-to-moderate interstitial fibrosis, leading to a dilation and systolic impairment of the left ventricle not attributable to abnormal loading conditions or coronary artery disease.^6^ Additionally, the BAG3 protein can influence myocyte contractility in adult cardiac myocytes as it is localized in the sarcolemma and T-tubules to modulate myocyte contraction in response to β-adrenergic receptor signaling,^12^ thereby playing an important role in the regulation of cardiac contractile function.^12^

The prevalence of *BAG3* DCM has been reported to be between 2.3%–3.6%, with more men than women developing adverse cardiac events, in particular heart failure.^13^ However, arrhythmias are not considered an adverse event. There have been cases reported that support the notion that *BAG3* mutation induces myofibrillar myopathies and polyneuropathy due to myofibrillar degeneration in young patients.^8^ We recognize that there is a possibility of peripheral neuropathy being a chance finding. Further follow-up may indicate a greater motor involvement.

In this case, it is likely that cardiac remodeling in the setting of genetic predisposition to DCM is another factor driving the onset of polymorphic VT presenting with presyncope. However, there are no reported cases of polymorphic VT with a *BAG3* exon 3 variant mutation. Furthermore, the discovery of the *BAG3* variant demonstrates a novel therapeutic target in the treatment and prevention of DCM and potential heart failure and a means to enhance cardiac performance and reduce arrhythmogenesis. Undoubtedly, the finding of this variant may be coincidental. Other family members of our patient have not been screened for this variant. The patient’s risk of sudden death also cannot be defined based on existing criteria.

## Conclusions

This case report illustrates the value of repeat monitoring if symptoms are persistent. The burden of ventricular arrhythmias can vary up to 7 times with repeat monitoring.^14^ It also illustrates the need for continued investigation if tachyarrhythmias persist despite correction of the presenting possible mechanism. In this case, the persistence of non-sustained symptomatic VT despite correction of the RCA anomaly prompted further investigation though scarring because pre-existing ischemia was a possibility. Genetic testing for a primary electrical disorder should be considered with persistent VT. Genetic testing in this case was significant for identification of a *BAG3* genetic variant. Although this is not reported to be associated with recurrent ventricular arrhythmias, it has been associated with DCM. We do recognize that it is possible that the variant may be a chance finding; it certainly emphasizes the need for genetic testing in patients where the cause of recurrent VT is not well defined. Routine testing for the *BAG3* variant cannot be recommended on the basis of a single case report, but it merits further investigation.
